# Antimicrobial resistance, plasmid, virulence, multilocus sequence typing and pulsed-field gel electrophoresis profiles of *Salmonella enterica* serovar Typhimurium clinical and environmental isolates from India

**DOI:** 10.1371/journal.pone.0207954

**Published:** 2018-12-12

**Authors:** Priyanka Jain, Sudhanthiramani Sudhanthirakodi, Goutam Chowdhury, Sangeeta Joshi, Shalini Anandan, Ujjwayni Ray, Asish Mukhopadhyay, Shanta Dutta

**Affiliations:** 1 Division of Bacteriology, Indian Council of Medical Research-National Institute of Cholera and Enteric Diseases, Kolkata, West Bengal, India; 2 Department of Microbiology, All India Institute of Hygiene and Public Health, Kolkata, West Bengal, India; 3 Hospital Infection Control, Manipal Hospital, Bangalore, Karnataka, India; 4 Department of Clinical Microbiology, Christian Medical College, Vellore, Tamil Nadu, India; 5 Department of Microbiology, Apollo Gleneagles Hospitals, Kolkata, West Bengal, India; Cornell University, UNITED STATES

## Abstract

*Salmonella enterica* serovar Typhimurium (*S*. Typhimurium) is a common serovar associated with non-typhoidal salmonellosis globally. However, there is insufficient data on molecular characterization of *S*. Typhimurium isolates from India. This study was undertaken to determine the antimicrobial resistance (AMR), plasmid, virulence profiles and molecular subtypes of *S*. Typhimurium Indian isolates (n = 70) of clinical and environmental origin isolated during 2010–2017. Antimicrobial susceptibility and minimum inhibitory concentrations were determined by disc diffusion and E-test methods respectively. Plasmid extraction was done following standard protocol. AMR genes, virulence genes and plasmid incompatibility types were detected by PCR; Pulsed-field gel electrophoresis (PFGE) and multi-locus sequence typing (MLST) were used for molecular subtyping. Majority (57%) of the study isolates was pan susceptible; five AMR profiles were observed among the resistant (43%) isolates. AMR was significantly (*p* = 0.004) associated with extra-intestinal isolates than intestinal isolates.The class 1 integron and plasmid-mediated quinolone resistance genes (*qnrB1*, *qnrS1*) in the resistant isolates were transferable by conjugation. Plasmids (≥1) ranging from 1.9 to 254kb size and of IncFIIS and/or FIB type were found in most isolates. A total of 39 pulsotypes by PFGE and four sequence types by MLST like ST36 (55.7%), ST19 (32.9%), ST313 (10%) and ST213 (1.4%) were observed. ST36 and ST19 were found circulating in both clinical and environmental host, while ST313 isolates had an exclusive clinical origin. All ST19 isolates (100%) were drug-resistant, while isolates belonging to ST313 (100%), ST213 (100%) and ST36 (82%) were pan susceptible. The virulence plasmid (VP) genes (*spvB- spvC*) were present in all genotypes except ST36. The VP was significantly (*p*<0.001) associated with extra-intestinal than intestinal isolates. Some environmental and clinical isolates were clonal indicating their zoonotic transmission. Knowledge on the molecular subtypes and AMR profiles of locally prevalent *Salmonella* serotypes is important for effective control of spread of resistant organisms. The MLST of *S*. Typhimurium isolates and its association with AMR, virulence profiles was not reported earlier from India.

## Introduction

Non-typhoidal *Salmonella* (NTS) infections represent a major zoonotic public health problem worldwide. *Salmonella enterica* serovar Typhimurium (*S*. Typhimurium) is the second most common NTS serotype, after *S*. Enteritidis causing human infections across the globe [1, 2]. It mostly causes self-limiting gastroenteritis but can lead to bacteraemia and focal extra-intestinal infections like meningitis, osteomyelitis, aortitis, septic arthritis especially in infants, elderly and immunocompromised individuals [2, 3]. Invasive *S*. Typhimurium infections have been more commonly reported from sub-Saharan African countries, where it is an important cause of morbidity and mortality in children under 5 years of age, second only to pneumococcal pneumonia [3, 4]. It has also been implicated in causing several foodborne outbreaks in adults [5, 6] as well as diarrhoeal and/or septicaemic outbreaks in neonates and pediatrics [7, 8].

Antimicrobial resistance in *S*. Typhimurium has been reported globally and of particular concern has been the emergence of multi-drug resistant (MDR; resistance to three or more classes of antimicrobials) strains which has complicated the currently available therapeutic options for these infections [3, 4, 9–12].

*S*. Typhimurium possess several virulence genes like *inv*A, *mgt*C, *sopB*, etc., which are clustered in the *Salmonella* pathogenicity islands (SPIs) on the chromosome and are involved in cellular invasion, intracellular survival and inflammation [13, 14]. The enterotoxin *(stn*) gene present outside the SPIs, mediates fluid and electrolyte secretion [14, 15]. Virulence plasmid (VP) of 90-95kb size is present in some strains of *S*. Typhimurium which help in bacterial multiplication within the reticulo-endothelial system of warm-blooded vertebrates. A 7.8kb region (*spvRABCD)* within the virulence plasmid confers the virulent phenotypes, of which the *spvB-C* gene is most important as it ADP-ribosylates the actin and thus destabilizes the cytoskeleton of eukaryotic cell. Other loci of the plasmid are involved in the biosynthesis of fimbriae (*pef*) and in serum resistance [16]. Virulence gene profiles of the local isolates could shed light on the virulence types of the organisms and predict clinical sequels of the disease.

Molecular sub-typing of bacteria is essential for surveillance of the pathogen in the environment, for determining the phylogenetic relatedness of the strains and for epidemiological investigations of outbreaks [17]. The two very popular molecular methods used in *S*. Typhimurium sub-typing include pulsed-field gel electrophoresis (PFGE) and multilocus sequence typing (MLST) [4, 10–12, 18]. Although, PFGE is considered as gold standard for *Salmonella* molecular sub-typing but rigorous standardization of protocol is necessary to ensure analysis reproducibility. On the other hand, the DNA sequence in MLST is unambiguous and data from different labs can be easily compared. Unlike PFGE, MLST can be used to study the evolutionary relationships of the isolates across the globe [10, 17].

Isolation of *S*. Typhimurium from humans, animals and food products has been frequently documented from the Indian subcontinent [19–29]. According to an article published from National *Salmonella* and *Escherichia* Centre, Kasauli, *S*. Typhimurium (24.7%) was found to be the most common NTS serotype isolated in India from all sources, based on the number of strains (n = 1795) randomly received by the Center for serotyping from various organizations of the country during 2001–2005 [24]. Although AMR, plasmid and virulence profiles of *S*. Typhimurium isolates from India were reported in the past [15, 20–23,25–29], data on molecular subtypes of the strains were rare, which has public health importance with respect to tracking and controlling the transmission of the *S*. Typhimurium isolates. This study was undertaken to address this issue by characterizing 70 *S*. Typhimurium isolates both clinical and environmental, with respect to their AMR, plasmid, virulence profiles, molecular subtypes by MLST and PFGE. Attempts have been made to study the association among the molecular subtypes, AMR profiles and virulence profiles of the isolates if there is any.

## Materials and methods

### Ethics statement

The present study was reviewed and approved by the Institutional Ethical Committee of National Institute of Cholera and Enteric Diseases (NICED), Kolkata.

### Bacterial isolates

Fifty-four clinical *S*. Typhimurium isolates submitted to Gastro-intestinal Tract Pathogen Repository (GTPR) of National Institute of Cholera and Enteric Diseases (NICED), Kolkata during 2010–2017 were included in this study. The GTPR unit of NICED receives isolates from various hospitals and institutes across the country for identification and confirmation of *Salmonella* by serotyping. The clinical isolates were recovered from patients admitted to various hospitals in Kolkata (n = 28), Bangalore (n = 14), Vellore (n = 9) and Dharwad (n = 3). These clinical isolates were further sub-classified into intestinal (n = 33) and extra-intestinal (n = 21) isolates. The intestinal isolates were obtained from human stool samples, while extra-intestinal isolates included those recovered from human blood (n = 15), pus (n = 4), synovial fluid (n = 1) and CSF (n = 1). We also included 16 environmental isolates of *S*. Typhimurium from our earlier study [29], for comparison with clinical isolates. The environmental isolates were recovered from poultry meat (n = 12), goat meat (n = 2), poultry cloacal swab (n = 1) and cattle rectal swab (n = 1) by random sampling of livestock and foods of animal origin for NTS in and around Kolkata during 2012–2013 [29].

The isolates stored in 50% glycerol in -80°C were revived by inoculation into Luria Bertini broth (Difco, Maryland, USA). The isolates were biochemically and serologically confirmed as *S*. Typhimurium by slide and tube agglutination using *Salmonella* poly- and monovalent O and H antisera (Denka Seiken Pvt. Ltd, Tokyo, Japan) using standard protocol [30, 31].

### Antimicrobial susceptibility testing

Antimicrobial susceptibility test of the study isolates was done following Kirby-Bauer disc diffusion method on Mueller Hinton agar (Merck, Darmstadt, Germany) using 15 antibiotic discs (BD BBL, Maryland, USA): ampicillin (A), chloramphenicol (C), tetracycline (T), co-trimoxazole (Q), nalidixic acid (Na), ciprofloxacin (Cip), ofloxacin (Ofx), gentamicin (Gm), amikacin (Ak), streptomycin (S), cefotaxime (Ctx), ceftazidime (Caz), ceftriaxone (Cro), aztreonam (Atm) and amoxycillin-clavulanic acid (Amc). Minimum Inhibitory Concentrations (MICs) of the antimicrobials for the resistant isolates were determined by E-test (AB Biodisk, Solna, Sweden). Results were interpreted following Clinical and Laboratory Standards Institute guidelines (CLSI) [32]. *Escherichia coli* ATCC 25922 was used as control.

### Determination of AMR genes, integrons and their transferability

All study isolates were screened by PCR for presence of AMR genes like *catA and cmlA* (chloramphenicol resistance); *tetA* and *tetB* (tetracycline resistance); *dfrIa*, *dfrVII*, *dfrXII*, *sul1*, *sul2* and *sul3* (co-trimoxazole resistance) and *aadA1* and *aadA2* (streptomycin resistance). Quinolone resistant isolates were screened for presence of any chromosomal mutations in the quinolone-resistance determining regions (QRDRs) of *gyrA*, *gyrB*, *parC* and *parE* genes and also for plasmid mediated quinolone resistance (PMQR) genes *qnrA*, *qnrB*, *qnrS*, *aac(6’)-Ib-cr* and *qepA*. Presence of class 1 integron (*int1*), its variable region containing the gene cassettes and *qacEΔ1* gene at 3’ conserved segment (CS) was also determined. All PCRs were performed using published primer sequences and PCR conditions [33–36]. Each PCR was followed by direct sequencing of the PCR product using 3730 DNA Analyzer (Applied Biosystems, Foster City, CA, USA). Sequences obtained were analyzed by comparison with the National Centre for Biotechnology Information (NCBI) database sequences using BLAST program.

Conjugation experiment was attempted by broth mating procedure in Tryptic Soy broth (Difco), using sodium azide resistant *E*. *coli* J53 as the recipient strain and resistant *S*. Typhimurium study strains as donors. Transconjugants were selected as lactose-fermenting colonies of *E*. *coli* on MacConkey agar supplemented with sodium azide (100 μg/ml) and one of the following antibiotics: chloramphenicol (16μg/ml), tetracycline (16μg/ml), streptomycin (50μg/ml) or ciprofloxacin (0.06μg/ml) [37, 38]. The transfer of AMR in transconjugants was checked by disc diffusion, MIC by E-test and presence of respective resistance genes by PCR.

### Plasmid extraction and incompatibility typing

Plasmid DNA of the study isolates and transconjugants were extracted following Kado and Liu method [39]. Plasmids were electrophoretically separated on 0.8% agarose gel at 70V for 3 hours in Tris-acetic acid-EDTA buffer. Gel was stained with ethidium bromide (0.5μg/ml), visualized under UV-transilluminator and photographed using gel documentation system (Bio-Rad, California, USA). The plasmid size was determined by Quantity One software version 4.5 (BioRad) using plasmid molecular weight markers *E*. *coli* V517 and *Shigella flexneri* YSH6000 as references. The incompatibility types of the plasmids were determined by PCR using published primers [40].

### Determination of virulence determinants

In this study, presence of five SPIs were investigated by targeting the following gene determinants: the *invA* gene for SPI-1 (helps in invasion), the *ssaQ* gene for SPI-2 (encodes type III secretion protein), the *mgtC* gene for SPI-3 (mediates intramacrophage survival), the *spi4D* gene for SPI-4 (encodes type I secretion protein) and the *sopB* gene for SPI-5 (mediates inflammation and chloride secretion). In addition, the VP gene (*spvB-spvC*), plasmid encoded fimbriae (*pef*) gene and enterotoxin (*stn*) gene were also determined by PCR, using published primer sequences and PCR condition [15, 33, 41].

### MLST

MLST was performed by PCR amplification followed by sequencing of the seven housekeeping genes: *aroC*, *dnaN*, *hemD*, *hisD*, *purE*, *sucA*, and *thrA* as described earlier [42]. Sequences obtained were submitted to the MLST database (https://enterobase.warwick.ac.uk/species/senterica/allele_st_search) to assign sequence type (ST) to each study isolate based on the set of alleles derived from the aforesaid seven loci.

### PFGE

PFGE of *Xba*I digested genomic DNA of the study isolates was performed, using CHEF DRIII (Bio-Rad), following the PulseNet standard protocol [43]. *S*. Braenderup H9812 was used as reference strain. The PFGE profiles were analyzed using FP Quest software version 4.5 (Bio-Rad). The extent of homology was determined by Dice coefficient with 1.5% optimization and tolerance levels, and clustering of bands was done by Unweighted Pair Group Method with Arithmetic mean (UPGMA). Based on PFGE profiles, one PFGE pulsotype (PP) number was assigned to each isolate. The isolates with identical PFGE patterns (Dice coefficient of similarity of 100%) were described as genetically indistinguishable; isolates with PFGE patterns differing by three or less bands (Dice coefficient of similarity of >80%) were designated as related and grouped under one cluster [44].

### Statistical analysis

χ2 test was used for statistical analysis. *p*-value less than 0.05 were considered statistically significant.

## Results

### Antimicrobial resistance profiles and MICs

Forty (57.1%) of 70 *S*. Typhimurium study isolates were pan susceptible to all antimicrobials tested, while thirty (42.9%) isolates showed resistance to at least one antimicrobial. The percentage of resistance to each antimicrobial is shown in Table 1. Five resistance profiles were observed among the resistant isolates: Na (n = 15), CTQSNa (n = 9), TNa (n = 4), TQ and T (n = 1 each). The MICs of each antimicrobial is shown in Table 2. None of the isolates showed resistance to higher generation antibiotics like fluoroquinolones and third generation cephalosporins.

**Table 1:** Percentage of antimicrobial resistance in *S*. Typhimurium isolates (n = 70) from India.

Antibiotic (potency)	Number (%)			Total (n = 70)
	Extra-intestinal (n = 21)	Intestinal (n = 33)	Environmental (n = 16)	
**Ampicillin (10μg)**	-	-	-	-
**Cloramphenicol (30μg)**	3 (14.3)	2 (6.1)	4 (25.0)	9 (12.9)
**Tetracycline (30μg)**	4 (19.0)	7 (21.2)	4 (25.0)	15 (21.4)
**Co-trimoxazole (25μg)**	4 (19.0)	2 (6.1)	4 (25.0)	10 (14.3)
**Nalidixic acid (30μg)**	13 (61.9)	8 (24.2)	7 (43.8)	28 (40.0)
**Ciprofloxacin (5**μ**g)**^a^	14 (66.7)	9 (27.3)	7 (43.8)	30 (42.9)
**Ofloxacin (5**μ**g)**^a^	14 (66.7)	9 (27.3)	7 (43.8)	30 (42.9)
**Amikacin (30μg)**	-	-	-	-
**Gentamicin (10μg)**	-	-	-	-
**Streptomycin (10μg)**	3 (14.3)	2 (6.1)	4 (25.0)	9 (12.9)
**Ceftazidime (30μg)**	-	-	-	-
**Cefotaxime (30μg)**	-	-	-	-
**Ceftriaxone (30μg)**	-	-	-	-
**Aztreonam (30μg)**	-	-	-	-
**Amoxycillin-clavulanic acid (30μg)**	-	-	-	-
**Resistance to ≥1 antimicrobial**	14 (66.7)	9 (27.3)	7 (43.8)	30 (42.9)
**Resistance to ≥3 antimicrobial (MDR)**	3 (14.3)	2 (6.1)	4 (25.0)	9 (12.9)

^a^Isolates showed intermediate susceptibility

MDR, multi-drug resistance, -, no resistance

**Table 2:** Antimicrobial resistance profile, minimum inhibitory concentration and antimicrobial resistant genes present in drug resistant *S*. Typhimurium isolates (n = 30).

Sl. No.	AMR-profile	Source of Isolation			MIC in μg ml^-1^							Antimicrobial resistant genes				Mutation in the QRDR of *gyr*A gene	PMQR gene	Size and gene cassette of class 1 integron
		Extra-intestinal	Intestinal	Environmental	C	T	Q	S	Na	Cip	Ofx	C	T	Q	S			
1.	Na (n = 15)	10	2	3					>256	0.125–0.5	0.25–0.75					Asp87-Tyr [GAC→TAC] (n = 14) and Ser83-Phe [TCC→TTC] (n = 1)	Absent	Absent
2.	CTQSNa (n = 9)	3	2	4	64->256	96->256	>32	64	>256	0.125–0.5	0.25–0.75	*cmlA1*	*tetA*	*dfrA12*, *sul3*	*aadA1*, *aadA2*	Asp87-Tyr [GAC→TAC]	Absent	7kb, *dfrA12*-*orfF*-*aadA2*-*cmlA1*-*aadA1*
3.	TNa (n = 4)	0	4	0		256			>256	0.5	0.75		*tetB*			Ser83-Tyr [TCC→TAC]	Absent	Absent
4.	TQ (n = 1)	1	0	0		>256	>32		16	0.38	0.75		*tetB*	*dfrA1*, *sul1*		Absent	*qnrB1*	1.2kb, *dfrA1*-*orfC*
5.	T (n = 1)	0	1	0		96			16	0.38	0.75		*tetA*			Absent	*qnrS1*	Absent

Asp, aspartate; Tyr, tyrosine; Ser, serine; Phe, phenylalanine

### Antimicrobial resistance genes, integrons and their transferability

Presence of AMR genes, class 1 integron and chromosomal mutations associated with Na resistance, found among the study isolates is shown in Table 2. Nalidixic acid resistance in the study isolates was associated with single mutations in the QRDR of *gyrA* gene at codon 87 or 83. No mutations were detected in *gyrB*, *parC* or *parE* genes. The PMQR gene *qnrB1* and *qnrS1* were found in two isolates (Table 2).

Among the various AMR genes found in the study isolates, the class 1 integron, *tetA*, *qnrB1* and *qnrS1* genes were transferable to the susceptible recipient strains by conjugation experiment. But the *tetB* genes were not transferable.

### Plasmid profile and typing

Fifty-nine (84.3%) of 70 *S*. Typhimurium study isolates harboured one to six plasmids with molecular size ranging from 1.9 to 254kb. Presence of a single plasmid was seen in 28 (47.5%) isolates, while 21 (35.6%) isolates possessed two plasmids and 10 (16.9%) isolates harbored more than two plasmids. Overall, a total of 26 plasmid profiles (A-Z) was found among the study isolates.The distribution of plasmid profiles among 59 study isolates is shown in Table 3. Plasmid typing revealed presence of IncFIIS (n = 49) and FIB (n = 38) plasmids. Plasmids of four isolates remained untypable (Table 3). Eleven isolates (5 intestinal and 6 environmental) were without plasmid.

**Table 3:** Distribution of plasmid profiles (with decreasing order of large plasmid) and incompatibility types in *S*. Typhimurium isolates (n = 59), India and its association with AMR-profile.

Profile	Approx. size in kb	Total no. of isolates	Distribution of isolates			Plasmid type			AMR-profile
			Extra-intestinal	Intestinal	Environmental	FIIS	FIB	Untypable	
A	254^a^, 130	9	3	2	4	+	+	-	CTQSNa
B	254, 21.2, 10.4, 2.9, 2.7, 2.3	1	0	1	0	+	+	-	Pan susceptible
C	234.5^a^, 23.9, 11.1, 2.8, 2.1	1	1	0	0	-	-	+	TQ
D	180, 98.5	3	0	3	0	+	-	-	Pan susceptible
E	155.8^a^, 4.6, 4.3, 3.3	1	0	1	0	-	+	-	T
F	155.8, 18.9, 11.1, 9.7, 5.9	1	0	1	0	-	+	-	TNa
G	155.8, 11.1, 9.7	1	0	1	0	-	+	-	TNa
H	155.8, 11.1, 9.7, 5.9, 5.2	1	0	1	0	-	+	-	TNa
I	155.8, 9.7, 5.9, 5.2	1	0	1	0	-	+	-	TNa
J	155.8, 5.9, 5.2, 4.7, 4.2	1	0	1	0	-	+	-	Pan susceptible
K	130	3	2	1	0	+	+	-	Na/pan susceptible
L	130, 2.5	1	0	1	0	+	+	-	Pan susceptible
M	108.4	9	5	2	2	+	+	-	Na/pan susceptible
N	108.4,4.9	1	1	0	0	+	+	-	Na
O	108.4, 3.2	1	1	0	0	+	+	-	Na
P	108.4, 4.7, 3.2	1	0	0	1	+	+	-	Na
Q	108.4, 2.5	2	2	0	0	+	+	-	Pan susceptible
R	98.5	13	1	10	2	+	-	-	Pan susceptible
S	98.5, 45.4	1	1	0	0	+	+	-	Na
T	98.5, 5.2	1	0	1	0	+	-	-	Pan susceptible
U	53.3, 51.4	1	1	0	0	+	+	-	Pan susceptible
V	51.4	1	1	0	0	+	+	-	Pan susceptible
W	45.4	1	1	0	0	+	+	-	Na
X	45.4, 11.1, 2.8, 2.1, 1.9	1	1	0	0	-	-	+	Na
Y	24.5	1	0	1	0	-	-	+	Pan susceptible
Z	9.1, 3.4	1	0	0	1	-	-	+	Pan susceptible

^**a**^Plasmids transferable by conjugation

+, present; -, absent

### Virulence gene profile

SPI-associated genes (*invA*, *ssaQ*, *mgtC*, *spi4D*, *sopB)* and the enterotoxin (*stn*) gene were uniformly present in all 70 (100%) study isolates. However, the VP encoded *spvB-C* and *pef* genes were present in only thirty-one (44.3%) isolates.

### MLST genotypes

The 70 study isolates were assigned to four sequence types (ST) by MLST: ST36 (n = 39, 55.7%), ST19 (n = 23, 32.9%), ST313 (n = 7, 10%) and ST213 (n = 1, 1.4%) (Table 4). The MLST data of the study strains has been submitted and is available at the MLST database (http://enterobase.warwick.ac.uk/species/senterica/search_strains).

**Table 4:** Molecular characteristics of prevalent MLST genotypes of *S*. Typhimurium isolates (n = 70) from India.

Sl. No.	Attributes of *S*. Typhimurium isolates	Number (%)				Total (n = 70)
		ST36 (n = 39)	ST19 (n = 23)	ST313 (n = 7)	ST213 (n = 1)	
1.	**Source of Isolation**					
	**Extra-intestinal**	4 (10.3)	12 (52.2)	5 (71.4)	0 (0.0)	21 (30.0)
	**Intestinal**	26 (66.7)	4 (17.4)	2 (28.6)	1(100.0)	33 (47.1)
	**Environmental**	9 (23.1)	7 (30.4)	0 (0.0)	0 (0.0)	16 (22.9)
2.	**AMR- profile**					
	**Pan susceptible**	32 (82.1)	0 (0.0)	7 (100.0)	1(100.0)	40 (57.1)
	**Na**	1 (2.7)	14 (60.9)	0 (0.0)	0 (0.0)	15(21.4)
	**CTQSNa**	0 (0.0)	9 (39.1)	0 (0.0)	0 (0.0)	9 (12.9)
	**TQ**	1 (2.7)	0 (0.0)	0 (0.0)	0 (0.0)	1 (1.4)
	**TNa**	4 (10.3)	0 (0.0)	0 (0.0)	0 (0.0)	4 (5.7)
	**T**	1 (2.7)	0 (0.0)	0 (0.0)	0 (0.0)	1 (1.4)
3.	**Virulence gene profile**					
	***stn*, *invA*, *ssaQ*, *mgtC*, *spi4D*, *sopB* (chromosomal genes)**	39 (100.0)	23 (100.0)	7 (100.0)	1(100.0)	70 (100.0)
	***spvB*, *spvC*, *pef* (virulence plasmid genes)**	0 (0.0)	23 (100.0)	7 (100.0)	1(100.0)	31 (44.3)
4.	**Plasmid profile and incompatibility type**					
	**Presence of plasmid**	28 (71.8)	23 (100.0)	7 (100.0)	1(100.0)	59 (84.3)
	**Number of plasmids**	1–5	1–3	1–6	1	1–6
	**Size range in kb (approx.)**	1.9–234.5	3.2–254	2.3–254	108.4	1.9–254
	**No. of plasmid profiles**	14 (C-K, R, T, X-Z)	8 (A, K, M-P, S, W)	6 (B, L, M, Q, U, V)	1 (M)	26 (A-Z)
	**IncFII**_**S**_ **and IncFIB**	1 (2.6)	23 (100.0)	7 (100.0)	1 (100.0)	32 (45.7)
	**IncFII**_**S**_	17 (43.6)	0 (0.0)	0 (0.0)	0 (0.0)	17 (24.3)
	**IncFIB**	6 (15.4)	0 (0.0)	0 (0.0)	0 (0.0)	6 (8.6)
	**Untypable plasmid**	4 (10.3)	0 (0.0)	0 (0.0)	0 (0.0)	4 (5.7)
5.	**PFGE profile**					
	**Number of pulsotype**	26 (P3, P11, P15, P16-P30, P32-P39)	9 (P1. P2, P4-P10)	3 (P12-P14)	1 (P31)	39
	**Similarity percentage**	84.7%	83.5%	91.6%	-	

### PFGE analysis

**Fig 1:**
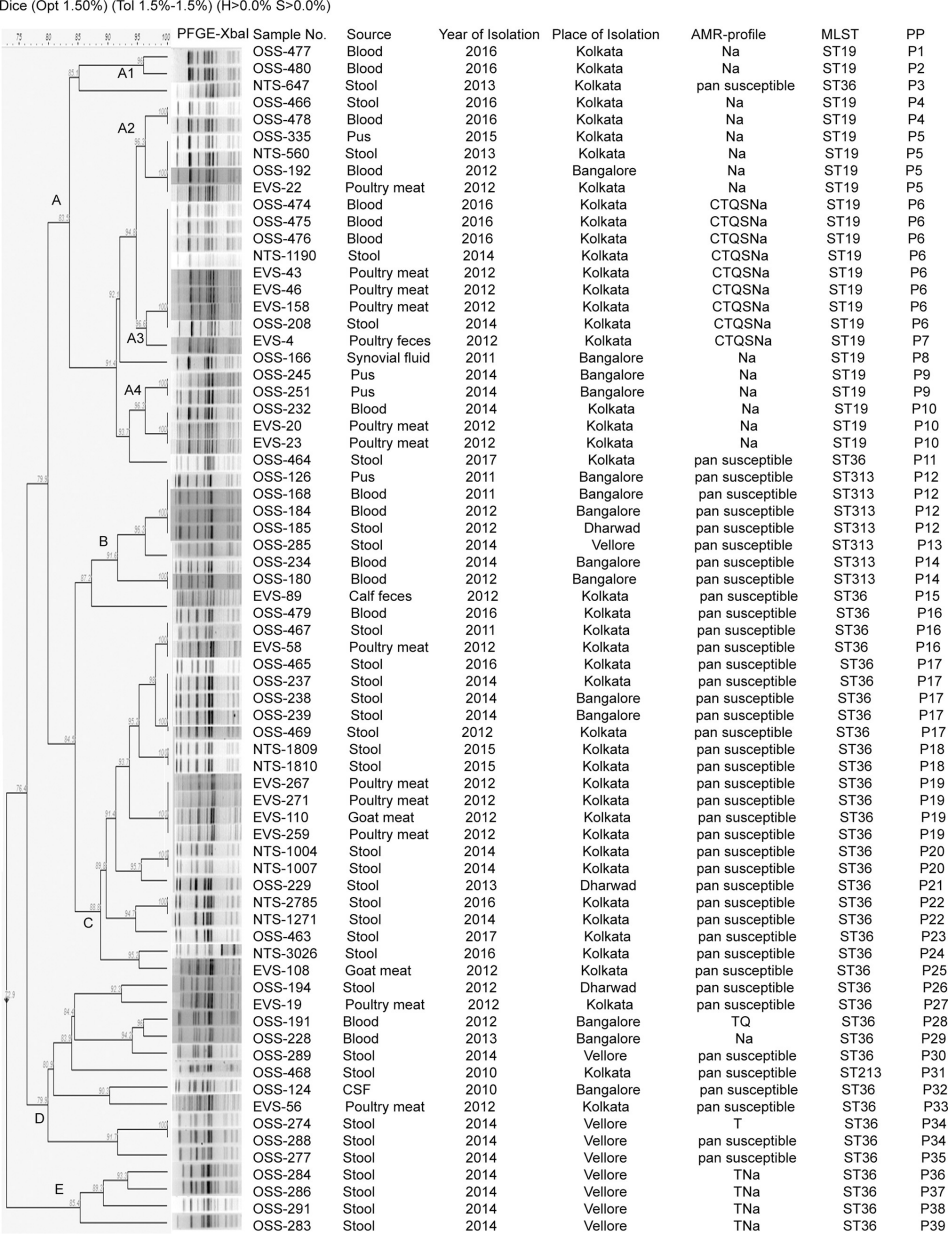
Pulsed-field gel electrophoresis (PFGE) profiles of *Xba*I digested DNA of *S*. Typhimurium isolates, India by cluster analysis. Band comparison was performed using Dice coefficient with 1.50% optimization (Opt) and 1.5% position tolerance (Tol). H, minimum height; S, minimum surface.

PFGE of 70 *S*. Typhimurium isolates generated 39 pulsotypes (P1-P39) which were grouped under five clusters (A-E) and showed similarity coefficient of 72.9% (Fig 1). Cluster A primarily comprised the drug-resistant ST19 isolates (n = 23) with nine pulsotypes (P1, P2, P4-P10) showing 83.5% homology. Four sub-clusters (A1-4) were observed within cluster A. The Na resistant isolates were grouped under A1 (n = 2; P1- P2), A2 (n = 6; P4- P5) and A4 (n = 5; P9-P10) and the isolates with CTQSNa profile formed sub-cluster A3 (n = 9; P6-P7). Cluster B consisted of pan susceptible ST313 isolates (n = 7) showing three pulsotypes (P12-P14) and 91.6% similarity. Isolates of ST36 subtype were diverse and showed 26 different pulsotypes (P3, P11, P15, P16-P30, P32-P39), majority being distributed in clusters C, D and E. The ST36 isolates of cluster C (n = 22; P16-P25) were pan susceptible showing 88.8% similarity. Isolates of cluster D (n = 10) were 79.9% similar and showed four different AMR profiles: pansusceptible, Na, TQ and T. The ST36 isolates (n = 4; P36-P39) with TNa profile formed cluster E and were 85.4% related.

## Discussion

This is a descriptive study, wherein 70 *S*. Typhimurium clinical and environmental isolates of 2010–2017 period were characterized with respect to their AMR, virulence and plasmid profiles; molecular subtypes by MLST and PFGE to provide an insight into the epidemiology of *S*. Typhimurium in Indian perspective. Despite the limited number of samples analysed, the phenotypes/genotypes found in this study make the investigation of pivotal importance in the background of epidemiology of *Salmonella*.

In this study 57.1% of the isolates were pan susceptible, while 42.9% isolates showed resistance to ≥1 antimicrobial. This finding was in tandem with the report of 65.9% pan susceptible *S*. Typhimurium isolates by National Antimicrobial Resistance Monitoring System for Enteric Bacteria (NARMS) in US during the period 2005–2014, but in contrast with the report of 32.1% pan susceptible *S*. Typhimurium isolates from Malaysia during 1970–2008 and 1.4% pan susceptible isolates from China during 2007–2011 [2, 11, 12]. Multidrug resistance (≥3 antimicrobial class) was found only in 12.9% study isolates, which was lower than those reported from Malaysia (50%), China (91.2%) and Egypt (70%) [11,12,45]. A decline in isolation of MDR *S*. Typhimurium in India was observed in past few decades from 95% in 1990s to 20% in 2008 [20, 22, 25].

The AMR in environmental study isolates (43.8%) was found to be more than clinical isolates (42.6%) but it was not significant statistically (*p* = 0.93). Since animals and food of animal origin are the source of infection in human, similar frequency of AMR from different sources is not unexpected. However, Cruchaga et al. in a study from Spain reported that resistance in *S*. Typhimurium isolated from animals was significantly higher (*p*<0.05) than those isolated from humans which was attributed to the use of antimicrobials as feed additives and in veterinary medicine [46]. In contrast Graziani et al. reported that *S*. Typhimurium human isolates (87%) in Italy were more resistant than animal isolates (81%) [47]. Among the clinical study isolates, resistance in *S*. Typhimurium of extra-intestinal origin was found to be significantly higher compared to isolates of intestinal origin (66.7% vs 27.3%, *p* = 0.004). This observation corroborated with the study conducted by NARMS which also reported that *S*. Typhimurium blood isolates were more likely to be associated with AMR than stool isolates [48].

Among the 15 antimicrobials tested, resistance to Na (40%) was predominantly observed among the *S*. Typhimurium isolates in this study. Although, similar Na resistance rate of 43% was reported by Taneja et al. from north-India (2002–2010), it was lower than those reported by Saha et al (96.3%) in an earlier study from Kolkata (1993–1996) [21, 27]. Nalidixic acid resistance rates ranging from 18–82.7% were reported from other countries of Asia [11, 12, 49]. Intermediate susceptibility to ciprofloxacin (MIC 0.125–0.5μg/ml) and ofloxacin (MIC 0.25–0.75μg/ml) were found in 30 (42.9%) study isolates, which is alarming as it has been associated with increased treatment failures with ciprofloxacin in invasive salmonellosis. Resistance to Cip was not obtained in this study, which was consistent with earlier reports from India (1993–2010) [21, 22, 25, 27].But sporadic cases of Cip resistant *S*. Typhimurium were reported from Assam (north-east India) and Vellore (south India) [23, 28]. Ciprofloxacin resistance (2–8%) was also reported from other Asian countries [11, 12, 45]. Unlike few reports from India documenting 11–20% resistance to Ctx in *S*. Typhimurium, susceptibiltity to Ctx and other third generation cephalosporins like Caz and Cro was found in this study [22, 25]. *S*. Typhimurium isolates resistant to third generation cephalpsporins have been reported frequently in countries like the US, Malaysia, China and Egypt in the past [2, 11, 12, 45]. Absence or low rate of resistance to antimicrobials like ampicillin, chloramphenicol, tetracycline and co-trimoxazole (Table 1) was observed in this study which suggested that these first line drugs might still be effective for treatment of salmonellosis. Diminution in resistance to first line drugs ranging from 60–100% in 1990–2001 to 20–45% in 2002–2010 was noted in *S*. Typhimurium Indian isolates [20–23, 25, 27].

The presence of AMR genes like *cmlA1*, *tetA*, *tetB*, *dfrA1*, *dfrA12*, *sul1*, *sul3*, *aadA1*, *aadA2* and class 1 integron as found in the study isolates, have been reported earlier in *S*. Typhimurium globally [10, 18, 25, 28, 33, 37, 45, 49, 50]. The Na resistant study isolates showed presence of a single point mutation (Asp87-Tyr or Ser83-Phe or Ser83-Tyr) in the QRDR of *gyrA* gene (Table 2). The muations Asp87-Tyr and Ser83-Phe are common in *S*. Typhimurium [34, 51]. Howerver, the muation Ser83-Tyr was not reported in *S*. Typhimurium earlier although it is frequently observed in other serovars like Montevideo, Hadar, Newport, Senftenberg and Enteritidis [34]. Thus our finding contradicts the report of Eaves et al., who showed that the substituting amino acid at codon 83 or 87 in *gyrA* differed between the *Salmonella* serovars [34]. Among the PMQR genes, *qnrB1* and *qnrS1* were present in two study isolates susceptible to Na (MIC 16 μg ml^-1^) but with intermediate susceptibility to Cip (MIC 0.38μg/ml) and Ofx (MIC 0.75μg/ml) (Table 2). PMQR genes (*qnr*S1, *qnr*B5, *qnr*B19, *aac(6’)-Ib-cr* and *qepA*.) have been reported earlier in Na susceptible *S*. Typhimurium isolates from Spain, Netherlands and US [36, 38, 52]. In India, *qnrB1* gene has been reported in Cip resistant *S*. Typhimurium strains [28].

Plasmid analysis revealed 26 profiles among 59 *S*. Typhimurium study isolates (Table 3). The number of plasmid profiles among isolates from different sources varied and were as follows: 15 profiles (A, B, D-M, R, T, Y) in intestinal isolates (n = 28), 13 profiles (A, C, K, M-O, Q-S, U-X) in extra-intestinal isolates (n = 21) and 5 profiles (A, M, P, R, Z) in environmental isolates (n = 10). Three plasmid profiles (A, M, R) were common among isolates of extra-intestinal, intestinal and environmental origin and included >50% of isolates. No correlation between plasmid profiles and AMR-profile was found except profile A, which was consistently found in all multi-drug resistant (CTQSNa-profile) isolates (Table 3). Of the two plasmids in profile A, the 254kb plasmid was transferable which carried the *cmlA1*, *tetA*, *dfrA12*, *sul3*, *aadA1*, *aadA2* and *int1* genes. Likewise, the transferable 234.5 kb plasmid (profile C) carried the *dfrA1*, *sul1*, *qacEΔ1*, *int1* and *qnrB1* genes & the transferable 155.8 kb plasmid (profileE) carried the *tetA* and *qnrS1*genes among the transconjugants. The localization of integron on transferable plasmids in this study aggravates the danger of dissemiation of AMR. Furthermore, the PMQR genes were co-transferred along with other AMR genes on same plasmid enabling simultaneous resistance to 2 classes of antimicrobials. It was noteworthy that the *tetB* genes in study isolates were not transferred by conjugation suggesting their localization on chromosomes or on non-conjugative plasmids. Plasmids of incompatibility type FIIS and FIB as found in this study and of other types like FIA-FIB, B/O, N, HI1, L/M and I1 have been described in *S*. Typhimurium [18, 25, 38, 40].

The presence of chromosomal virulence genes (*invA*, *ssaQ*, *mgtC*, *spi4D*, *sopB* and *stn*) in all study isolates was in tandem with previous reports [15, 26, 45, 53]. The VP associated *spvB/C* and *pef* genes were detected in 44.3% study isolates, which was lower compared to the earlier studies from north-east India (96%) and France (82%) [15, 53]. While the role of VP has been extensively studied in murine model, its relevance in human systemic infections is controversial. While some suggest that *spv* genes help in dissemination of *S*. Typhimurium from the intestine, others support the view that it is unessential for systemic infections. In this study the VP genes were significantly (*p*<0.001) associated with extra-intestinal isolates (n = 17/21, 81%) than intestinal isolates (n = 7/33, 21.2%). Earlier Heithoff et al. had also observed the presence of VP in all (100%) *S*. Typhimurium bacteraemic strains and in 66% gastrointestinal strains [54]. However Weisner et al., could not find any association of VP with systemic infection but they showed that it was significantly associated with human hosts than animal hosts [10]. Such finding was not observed in this study. The percentage of VP in clinical (n = 24/54, 44.4%) and environmental (n = 7/16, 43.8%) isolates were not significant (*p* = 0.96). It is speculated that besides the VP, host factors like age and any kind of immunosuppresive therapy play an important role in the dissemination of *Salmonella* to bloodstream. In this study, 86% of patients with extra-intestinal infection had either some underlying disease conditions (malignancy, diabetes, HIV, perianal abscess or biliary atresia) or were infants with weak immunity (S1 Table). No relation between virulence genes and AMR profile was observed as VP genes were present in both resistant and pan susceptible isolates (Table 4).

*S*. Typhimurium study isolates were typed into 4 genotypes by MLST, with majority of the isolates grouped into ST36 (55.7%) followed by ST19 (32.9%), ST313 (10%) and ST213 (1.4%). The multilocus sequence types of *S*. Typhimurium in India were not reported earlier. All the four STs have been previously reported in *S*. Typhimurium [4, 10, 12, 18]. According to the MLST database (https://enterobase.warwick.ac.uk/species/senterica/allele_st_search), ST19 is the most common genotype of *S*. Typhimurium found across the globe. ST36 has been frequently reported from Asian (Taiwan, Malaysia, China, Thailand) and European (United Kingdom, France, Norway) countries. ST313 and ST213 have been predominant in Africa (Kenya, Malawi, Zimbabwe, Uganda, Ethopia) and Mexico respectively. Although ST313 and ST213 had limited geographical distribution in the past, but they have now spread to other continents due to immigration, transport and trade. An endemic genotype identified in Asia (China, Thailand) was ST34 [12], which was not observed in this study.

Some association between *S*. Typhimurium STs and source of isolation; STs and AMR profile; STs and VP were observed in this study (Table 4). While ST19 and ST36 were isolated from both clinical and environmental samples, ST313 were of human origin exclusively. A strong association of ST313 isolates with disease in humans due to adaptation to human host as a result of genome degradation was reported by Kingsley et al. [4]. The ST19 in this study was found to be significantly associated with extra-intestinal isolates (*p*<0.001), while ST36 was found to be significantly prevalent in intestinal isolates (*p*<0.0001). All ST19 isolates in this study were drug resistant and showed two types of AMR-profile (Na and CTQSNa), while ST313 (100%), ST213 (100%) and ST36 (82%) in this study were pan susceptible. The AMR profiles were associated with specific STs, thus particular ST could be proposed as predictors of AMR. Association of *S*. Typhimurium ST19 with drug resistance has been reported earlier [10, 12, 18]. In contrast to this study, earlier studies have reported high drug resistance in ST313, ST213 and ST36 isolates [4, 10, 12]. The VP associated genes (*spvB-C* and *pef*) were found in all ST19, ST313 and ST213 *S*. Typhimurium study isolates but was absent in all ST36 isolates. The presence of VP in ST19, ST213 and ST313 isolates were reported earlier [4, 10], although the presence/absence of VP in ST36 is not known. It was also noteworthy that all isolates of ST19 and ST313 genotypes harbored plasmids of both IncFIIS and FIB type. However, only 72% of ST36 isolates possessed plasmids which belonged to either FIIS or FIB (Table 4).

PFGE of study isolates identified 39 pulsotypes and five clusters (A-E) with 72.9% similarity co-efficient (Fig 1) suggesting non-clonality among the circulating *S*. Typhimurium Indian isolates. However the isolates within each cluster were closely related (similarity co-efficient of ≥80%). Earlier Rahman had also reported heterogeneity among *S*. Typhimurium isolates from India. Twelve PFGE patterens were observed among 17 human and environmental isolates [22]. With respect to the source of isolation, 14 pulsotypes were observed among extra-intestinal isolates, 24 pulsotypes among intestinal isolates and 10 pulsotypes among environmental isolates Clonality was found between few environmental and clinical isolates (P5, P6, P10, P16 in Fig 1), thus re-establishing their zoonotic transmission via food products of animal origin. Heterogeneity was observed within isolates with similar AMR-profile. For example, 8 patterns were observed in Na-resistant isolates (n = 15) and 4 patterns were observed in TNa-resistant isolates (n = 4).There was no overlapping of PFGE pulsotypes among *S*. Typhimurium with different AMR-profiles, except P34 which was observed in both pansusceptible and T-resistant isolate (Fig 1). PFGE clusters showed good correlation with ST and antimicrobial susceptibility phenoytype.

The limitation of this study was that we could characterize only those *S*. Typhimurium isolates that were submitted to the GTPR unit of NICED during 2010–2017. We could not get isolates from other parts of the country, the inclusion of which would have helped to get a clearer picture of the disease epidemiology of *S*. Typhimurium in India.

## Conclusions

The study provides a brief overview of the AMR, plasmid, virulence profiles as well as MLST and PFGE subtypes of *S*. Typhimurium isolates circulating in India. Majority of the isolates were susceptible to common antimicrobials. However, the high rate of resistance to Na and intermediate susceptibility to fluoroquinolones is alarming as these are the current drug of choice for treatment of salmonellosis. Isolation of *S*. Typhimurium carrying transferable resistance genes reinforces the need for an effective surveillance.The observed association between the molecular subtypes and AMR/virulence profiles of *S*. Typhimurium endemic Indian isolates might be useful in tracking the source of infection and thus control spread of resistant organisms. To the best of our knowledge, this is the first report on MLST of *S*. Typhimurium isolates from India.

## Supporting information

S1 Table Medical history of patients from whom *S.* Typhimurium were recovered. (DOCX)
