# Supplementary material for: Antimicrobial resistance, plasmid, virulence, multilocus sequence typing and pulsed-field gel electrophoresis profiles of Salmonella enterica serovar Typhimurium clinical and environmental isolates from India
Source: PLoS One. 2018 Dec 12;13(12):e0207954. doi: 10.1371/journal.pone.0207954 (PMC6291080; doi:10.1371/journal.pone.0207954)
Supplement: S1 Table — (DOCX) [file pone.0207954.s001.docx]

**S1 Table. Medical history of patients from whom *S*. Typhimurium were recovered.**

|  | **Age** | | | **Sex** | | **Underlying Disease condition** | | | | |
| --- | --- | --- | --- | --- | --- | --- | --- | --- | --- | --- |
|  | **≤5 years** | **5-60 years** | **>60**  **years** | **male** | **female** | **Diabetes** | **HIV** | **Malignancy** | **Malaria** | **others^a^** |
| **Extra-intestinal isolates (n=21)** | 5 (23.8) | 10 (47.6) | 6 (28.5) | 15 (71.4) | 6  (28.6) | 1(4.8) | 2 (9.5) | 9 (42.9) | 0 (0.0) | 4 (19.0) |
| **Intestinal isolates (n=33)** | 11 (33.3) | 18 (54.5) | 4 (12.1) | 11 (33.3) | 22 (66.7) | 0 (0.0) | 1 (3.0) | 0 (0.0) | 1 (3.0) | 2 (6.1) |

Values in parentheses are percentages

^a^others include osteomyelitis, perianal abscess, biliary atresia and liver cirrhosis
